# The 10-m crop type maps in Northeast China during 2017–2019

**DOI:** 10.1038/s41597-021-00827-9

**Published:** 2021-02-02

**Authors:** Nanshan You, Jinwei Dong, Jianxi Huang, Guoming Du, Geli Zhang, Yingli He, Tong Yang, Yuanyuan Di, Xiangming Xiao

**Affiliations:** 1 Key Laboratory of Land Surface Pattern and Simulation, Institute of Geographic Sciences and Natural Resources Research, Chinese Academy of Sciences, Beijing, 100101 China; 2 University of Chinese Academy of Sciences, Beijing, 100049 China; 3 College of Land Science and Technology, China Agricultural University, Beijing, 100083 China; 4 School of Public Administration and Law, Northeast Agricultural University, Harbin, 150030 China; 5 Department of Microbiology and Plant Biology, University of Oklahoma, Norman, OK 73019 USA

**Keywords:** Agriculture, Geography

## Abstract

Northeast China is the leading grain production region in China where one-fifth of the national grain is produced; however, consistent and reliable crop maps are still unavailable, impeding crop management decisions for regional and national food security. Here, we produced annual 10-m crop maps of the major crops (maize, soybean, and rice) in Northeast China from 2017 to 2019, by using (1) a hierarchical mapping strategy (cropland mapping followed by crop classification), (2) agro-climate zone-specific random forest classifiers, (3) interpolated and smoothed 10-day Sentinel-2 time series data, and (4) optimized features from spectral, temporal, and texture characteristics of the land surface. The resultant maps have high overall accuracies (OA) spanning from 0.81 to 0.86 based on abundant ground truth data. The satellite estimates agreed well with the statistical data for most of the municipalities (R^2^ ≥ 0.83, p < 0.01). This is the first effort on regional annual crop mapping in China at the 10-m resolution, which permits assessing the performance of the soybean rejuvenation plan and crop rotation practice in China.

## Abstract (summary)

**Table:** 

Measurement(s)	area of different crop types • area of cropland
Technology Type(s)	machine learning
Factor Type(s)	type of crop • year of data collection
Sample Characteristic - Environment	cultivated environment • cropland ecosystem
Sample Characteristic - Location	Northeast China • China

Machine-accessible metadata file describing the reported data: 10.6084/m9.figshare.13567526

## Background & Summary

Northeast China has become the increasingly important grain bowl for the country^1^; however, the cropping systems in this region has changed significantly year by year due to the crop rotation practice and soybean rejuvenation plan targeting sustainable agricultural production and relieving pressure on international trade of soybeans, respectively^2^. Quantitative information about the changes in the farming system is still unavailable, due to the lack of the annual crop maps, which impedes our understanding of cropland dynamics and underlying drivers of farming system changes.

With the convergence of the newly available moderate resolution satellite imagery, new algorithm developments, and cloud computing infrastructure, considerable progress has been made on crop mapping^3^. Country-wide operational crop mapping systems emerged, such as the Cropland Data Layer (CDL) of the US Department of Agriculture (USDA)^4^; the Agriculture and Agri-Food Canada’s Annual Crop Inventory (AAFC) in Canada^5^; and the Sen2Agri automated system for Europe and parts of Africa^3,6^. In China, however, this kind of platform is still unavailable which hamper the decision-making related to food security for the most populous country. Although Landsat images could provide more spatial details comparing to the previous efforts using coarse resolution MODIS data^7^, the 16-day revisit cycle could not easily disentangle different crop phonologies, thus limiting the accuracy of the resulting maps^2,8^. The Sentinel-2A/B (S2) satellites acquire images with a spatial resolution of 10-meters (blue, green, red, and NIR bands) and 20-meters (Red Edge 1, Red Edge 2, Red Edge 3, Red Edge 4, SWIR1, and SWIR2 bands), and together they provide images with a 5-day interval, which opens a completely new avenue for crop-specific monitoring at the parcel level. The spatial resolutions at 10-m to 20-m could depict individual fields in many regions^9^. The relatively short revisit cycle could provide more detailed phenological information related to individual crop types. Moreover, the crucial spectral wavelength domains included several red-edge bands, which may help discriminate rather subtle differences among morphologically similar crop types^10^. The red-edge bands of S2 have been proved to be effective to distinguish maize and soybean^11^. Therefore, it would be a priority to demonstrate the feasibility of all the S2 images on major crop mapping in Northeast China.

Despite abundant efforts in crop mapping, it is still challenging to map major crops annually in entire Northeast China. First, the absence of up-to-date field boundaries layers hampered the crop mapping, because other land covers (e.g. grass and trees) need to be pre-filtered. Second, the variation in crop spectrum and phenology over large scales would limit the classification accuracy^12^. The different climate, crop varieties, and management practices caused high intra-class variabilities of the crop spectrum and phenology in the entire region. Third, the frequency and dates of valid satellite observations largely differed across time and space due to the different satellite orbits, varied dates, and location of cloud contamination^3^. Irregular image time series cannot be directly used to develop classification models in most cases. Fourth, the effective classification features were not well documented when using high spectral, temporal, and spatial resolutions of satellite data (i.e. S2)^13^. The poor understanding of the feature performance would either omit important features or include irrelevant features. Both circumstances adversely affected the classification performance^7^. To deal with the challenges mentioned above, here we try to develop a new framework to generate annual crop maps. (1) We adopt a hierarchical approach to separate cropland mapping and crop type classification. A cropland mask was generated, and the crop type classification was followed within the cropland extent. (2) To alleviate the negative impacts of the spectral and phenological variability of a specific crop across space, we generated regionally independent classifiers by considering agro-climate zones (ACZs), which had regionally consistent cropping systems. (3) To obtain homogeneous time series and fill the data gaps, regular time series of S2 images was generated based on the interpolation and smoothing algorithms. (4) To avoid the Hughes effect (also known as the “curse of the dimensionality”) and save computing time^14^, we developed a sophisticated feature selection procedure to select optimal features from the huge size of S2-based feature candidates.

The objective of this study is to produce annual crop maps in Northeast China from 2017 to 2019 at 10-m spatial resolution using (1) a hierarchical mapping strategy, (2) agro-climate zone-specific random forest classifiers, (3) interpolated and smoothed S2 time series, and (4) optimized features from spectral, temporal, and texture information. All the available S2 images, Google Earth Engine (GEE) platform, and the random forest algorithm were used for crop mapping. Our consistent crop maps can be utilized to monitor crop dynamics and to assess the effects of land-use policies.

## Methods

### Study area

**Fig. 1:**
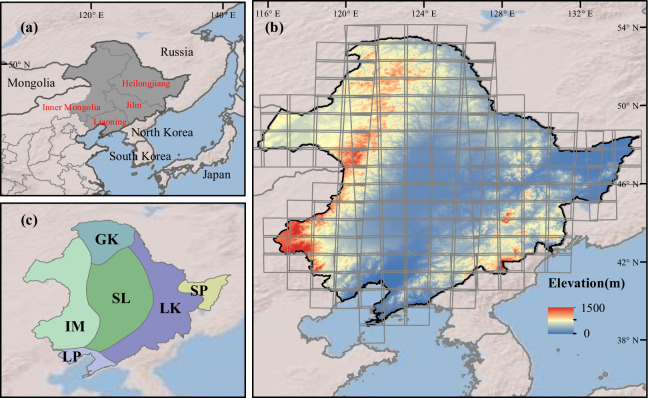
The location (**a**) and the topographical characteristic (**b**) of Northeast China, and the six agro-climate zones (ACZs) in Northeast China (**c**). The Sentinel-2 tiles covered Northeast China were showed in subplot b.

Our study area is the Northeast China (39° N**–**54° N, 115° E**–**135° E), including the Heilongjiang province, the Jilin province, the Liaoning province, and the four municipalities in eastern Inner Mongolia (Fig. 1a,b). Northeast China has an area of 1.2 million km^2^, about 13% of China’s territory. Northeast China spans six agro-climate zones (ACZs) according to the “the Regionalization of Agro-climate of China”^15^, including the North Greater Khingan (GK), the Sanjiang Plain (SJ), the Lesser Khingan and Changbai Mountains (LK), the Songliao Plain (SL), the Liaodong Peninsula (LD), and East Inner Mongolia (IM) (Fig. 1c). Annual accumulated air temperatures above 0 °C range from 2000–4200 °C·day, and the annual accumulated air temperatures above 10 °C vary from 1600–3600 °C·day^16^. Annual precipitation is concentrated in July and August, ranging from 500 to 800 mm. The number of frost-free days varies between 140 and 170 days^16^. As one of the most important food bowls in China, Northeast China occupies more than 15% of the total crop planting area in China^17^. The major crops are maize, soybean, and rice, and the sum of the planting area of these three crops exceeded 90% of the total crop planting areas in Northeast China. We did not identify wheat because the planting area of wheat only occupies about 0.4% of the total crop planting areas in the study area. Single cropping dominates Northeast China due to the accumulated temperature limit.

### Overview of the crop classification method

**Fig. 2:**
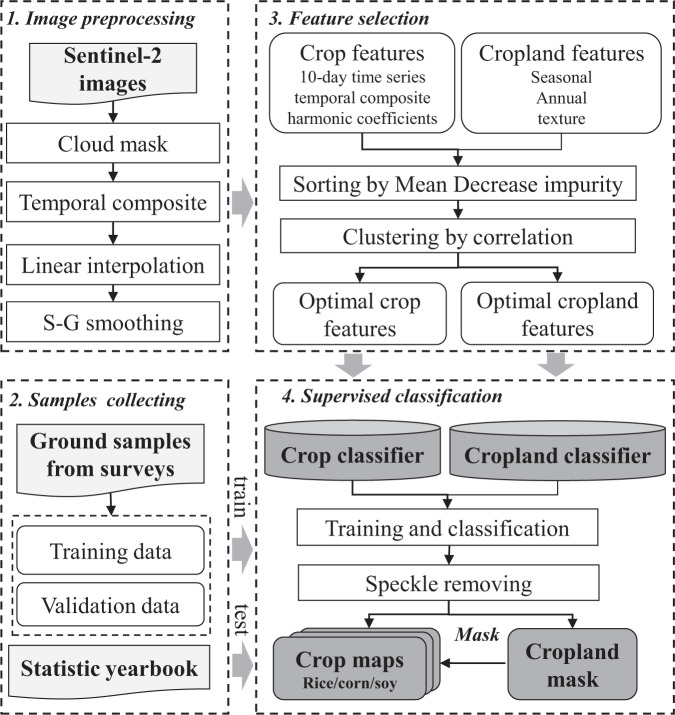
The workflow of the crop classification in the Northeast China.

We adopted the Random Forest (RF) algorithm and a sophisticated feature selection procedure to classify the cropland and crop types based on Google Earth Engine (Fig. 2). In each of the six ACZs and each of the three years, the crop type map was independently generated by three steps: (1) A stable cropland layer was generated to exclude the non-crop pixels. The cropland extent rarely changed in Northeast China during 2017–2019 due to the mature agricultural development and strict policies on cropland protection in Northeast China in recent years^15,18^. Therefore, only one cropland layer was produced during the three years. We conducted the binary classification (cropland vs non-cropland) based on the training samples, optimal cropland features, and random forest (RF) algorithm in the GEE. (2) Different crop types were classified within the cropland. We used optimal crop features as inputs to train the crop classifier (rice, maize, soybean, and other crops) based on the RF algorithm and then applied the classifier to S2 images. (3) A “despeckler” algorithm was utilized on the classification output to reduce speckle^19^. For the crop patches smaller than 0.1 ha, the output was updated via a circular kernel-based majority filter with a radius of 100 m. Most of the speckles disappeared in the resulting maps via the “despeckler” algorithm.

### Sentinel-2 images and pre-processing

We used Sentinel-2A/B (S2) Multi-Spectral Instrument (MSI) top-of-atmosphere (TOA) reflectance images (Level-1C) from 2017–2019, as the S2 surface reflectance (SR) data (Level-2A) in the study area before 2019 were not available at the Google Earth Engine (GEE) platform. Previous studies have proved the reliability of TOA reflectance on image classification because the relative spectral differences are the essential aspect^20^. Lots of recent efforts have used S2 TOA images to observe crops, such as the paddy rice mapping^21^, maize area and yield mapping^22^, sugarcane identification^23^, and cropping intensity monitoring^24^. The cloudy observations of the S2 TOA data were removed based on the adjusted cloud score algorithm^25^. Specifically, four bands (Aerosols, Blue, Green, and Red band) and two spectral indices (Normalized Difference Moisture Index (NDMI) and Normalized Difference Snow Index (NDSI)) were used to compute cloud score and detect cloud for S2 data, considering the fact that clouds are reasonably bright in the blue and cirrus bands, in all visible bands, and are moist. The adjusted cloud score algorithm could detect clouds more accurately than the QA60 quality assessment band^11^.

We further processed the time series data in the three steps: (1) 10-day composites were generated with the median values of the valid S2 observations; (2) data gaps were filled by the linear interpolation to achieve full coverages throughout the temporal domain^10^, and (3) 10-day time series data were smoothed by using the Savitzky-Golay (SG) filter^24^. In this study, we used the window size of 70 days (7 observations) and the 3rd order polynomial. Finally, we obtained regular cloud-free and gap-filled 10-day S2 time series (Fig. S1) .

Two types of spectral information were used for the cropland and crop type classification: (1) the reflectance of three spectral bands and (2) the value of seven spectral indices (Table 1). Three bands including Red Edge2 (RE2, 740.2 nm), Shortwave Infrared band1 (SWIR1, 1613.7 nm) and Shortwave Infrared band2 (SWIR2, 2202.4 nm) were utilized. Previous studies have reported the efficacy of SWIR1, SWIR2 and RE2 for the discrimination of the maize and soybean^11,26^. Seven commonly used spectral indices were obtained in particular: Normalized Difference Vegetation Index (NDVI)^27^, Enhanced Vegetation Index (EVI)^28^, Land Surface Water Index (LSWI)^29^, Normalized Differential Senescent Vegetation Index (NDSVI)^30^, Normalized Difference Tillage Index (NDTI)^31^, Red Edge NDVI (RENDVI) and Red Edge Position (REP)^3^. NDVI and EVI time series has been widely used to extract temporal features or phenological metrics of different crops^30,32^. LSWI could identify paddy rice and classify maize and soybean due to its high sensitivity to leaf water and soil moisture^26,29^. NDSVI is related to crop-specific responses to water content, and NDTI is an indicator of residue cover. These two indices had been used to develop phenology-based classification method to map corn and soybean^30^. RENDVI and REP, making use of the S2 Red Edge bands (around 704 nm, 740 nm and 783 nm), are particularly suitable for estimating canopy chlorophy II and nitrogen content^33^. Although they are critical for agriculture, the performance on crop classification remains under-recognized.

**Table 1:** The formulation of the seven spectral indices used in the study.

Indices	Formulation*	Reference
NDVI	${\rm{NDVI}}=\frac{{\rho }_{NIR}-{\rho }_{red}}{{\rho }_{NIR}+{\rho }_{red}}$	^28^
EVI	${\rm{EVI}}=2.5\times \frac{{\rho }_{NIR}-{\rho }_{red}}{{\rho }_{NIR}+6\times {\rho }_{red}-7.5\times {\rho }_{blue}+1}$	^29^
LSWI	${\rm{LSWI}}=\frac{{\rho }_{NIR}-{\rho }_{SWIR1}}{{\rho }_{NIR}+{\rho }_{SWIR1}}$	^30^
NDSVI	${\rm{NDSVI}}=\frac{{\rho }_{SWIR1}-{\rho }_{red}}{{\rho }_{SWIR1}+{\rho }_{red}}$	^31^
NDTI	${\rm{NDTI}}=\frac{{\rho }_{SWIR1}-{\rho }_{SWIR2}}{{\rho }_{SWIR1}+{\rho }_{SWIR2}}$	^32^
RENDVI	${\rm{RENDVI}}=\frac{{\rho }_{NIR}-{\rho }_{RE2}}{{\rho }_{NIR}+{\rho }_{RE2}}$	^3^
REP	${\rm{REP}}=\frac{705+35\times (0.5\times \left({\rho }_{RE3}+{\rho }_{red}\right)-{\rho }_{RE1})}{{\rho }_{RE2}-{\rho }_{RE1}}$	^3^

*ρ_*blue*_, *ρ*_*red*_, *ρ*_*RE1*_, *ρ*_*RE2*_, *ρ*_*RE3*_, *ρ*_*NIR*_, *ρ*_*SWIR1*_ and *ρ*_*SWIR2*_, are top-of-atmosphere (TOA) reflectance of Band 2 (blue, 496.6 nm (S2A)/492.1 nm (S2B)), Band 4 (red, 664.5 nm (S2A)/665 nm (S2B)), Band 5 (red edge 1, 703.9 nm (S2A)/703.8 nm (S2B)), Band 6 (red edge 2, 740.2 nm (S2A)/739.1 nm (S2B)), Band 7 (red edge 3, 782.5 nm (S2A)/779.7 nm (S2B)), Band 8 A (NIR, 864.8 nm (S2A)/864 nm (S2B)), Band 11 (SWIR1, 1613.7 nm (S2A)/1610.4 nm (S2B)) and Band 12 (SWIR2, 2202.4 nm (S2A)/2185.7 nm (S2B)) in the Sentinel-2 MSI sensor.

### Training and validation data

**Fig. 3:**
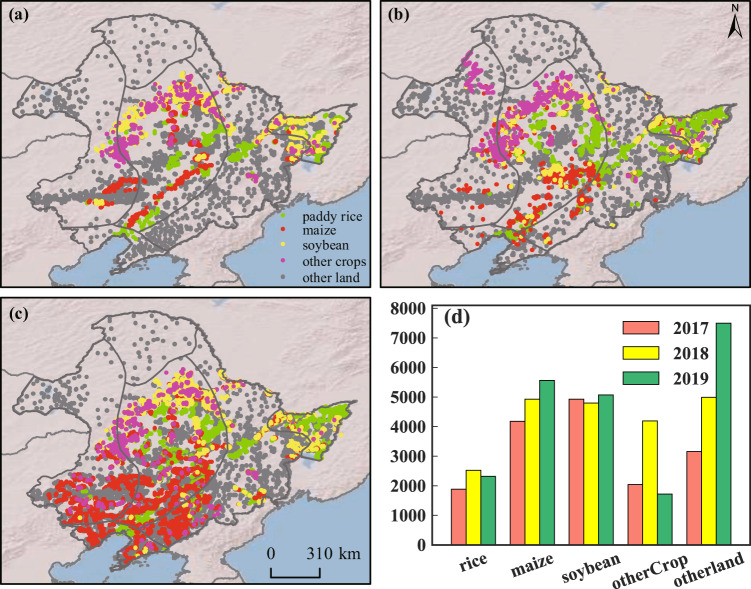
The distribution of the ground truth samples in 2017 (**a**), 2018 (**b**) and 2019 (**c**). The number of the ground truth samples in the three years was displayed in subplot d.

We collected ground samples from field surveys in three years (Fig. 3a–c). The location and the crop type of each sample were recorded using a mobile GIS device (an iPad equipped with a GIS software OvitalMap) in the field along the route. Other land cover types (e.g. grassland, wetland, forest, water body, and build-up) were also recorded. After the field surveys, all the ground samples were visually checked using high-resolution images in Google Earth and two S2 RGB composites, including the RGB composite (R: SWIR1, G: NIR, B: Red) from the mid-April to mid-June and the RGB composite (R: NIR, G: SWIR1, B: SWIR2) during early-July to late-August. The samples with obvious errors (such as incorrectly labeled the nature vegetations as crops) were excluded. The samples lied in the roads or the field boundaries were also removed. In addition, we added some non-cropland samples through visual interpretation on the high resolution image of Google Earth. Finally, we got a number of ground samples in the order of 16,187, 21,431, and 22,171 in 2017, 2018, and 2019 (Fig. 3d). In each year, the samples were randomly and equally divided into two parts, one part used for training and classification, another part used for accuracy evaluation.

### Feature selection

We used seasonal/annual spectral-temporal metrics and texture metrics to identify croplands (Table 2). The seasonal and annual spectral-temporal metrics could capture seasonal variations of land surface spectra^34,35^. According to the crop calendars of the main crops in Northeast China, we divided the entire growing season (Day Of Year(DOY):90–300) into three periods: the seeding stage (DOY:109–169), the growth stage (DOY:170–230), and the harvest stage (DOY:231–291)^36^. In each of the three periods, we obtained the medians of the three reflectance bands (i.e. RE2, SWIR1, and SWIR2) and seven spectral indices (i.e. NDVI, EVI, LSWI, NDSVI, NDTI, RENDVI, and REP)(Table 1). In the entire growing season, we calculated more metrics to depict spectral means and variances, including minimum, maximum, mean, standard deviation, amplitude, and the 5th, 25th, 50th, 75th, and 95th percentiles. We also included some texture measures given the homogeneous nature of cropland fields. The median values of NDVI observations in the crop seeding, growth, and harvest stage were used to calculate the texture measures. For each image, 18 texture features were calculated by a gray-level co-occurrence matrix (GLCM)^37,38^. Aiming at a greater number of observations available for the generation of cropland mask, the S2 images during 2017–2019 were merged to compute spectral-temporal and texture metrics. For example, all images in DOY 109–169 of three years were merged to compute seasonal metrics in the crop seeding stage. In total, 184 feature candidates were obtained to produce the cropland map.

**Table 2:** The summary of the feature candidates for cropland mask generation.

Group	Periods	Proxies	Metrics	# of features
Seasonal metrics	seeding stage, growth stage, harvest stage	RE2, SWIR1, SWIR2, NDVI, EVI, LSWI, NDSVI, NDTI, RENDVI, REP	median	30 (10 proxies /stage × 3 stages)
Annual metrics	growing season	RE2, SWIR1, SWIR2, NDVI, EVI, LSWI, NDSVI, NDTI, RENDVI, REP	min, max, mean, std, amplitude, and 5/25/50/75/95th percentile	100 (10 proxies /metric × 10 metrics)
Texture measures	seeding stage, growth stage, harvest stage	NDVI	gray-level co-occurrence matrix	54 (18 metrics/stage × 3 stages)

We employed three groups of feature candidates to discriminate the crop types (Table 3). (1) 10-day time series of the three reflectance bands (i.e. RE2, SWIR1, and SWIR2) and seven spectral indices (i.e. NDVI, EVI, LSWI, NDSVI, NDTI, RENDVI, and REP) in the growing season (DOY: 90–300) (Fig. S1); (2) three bands and seven indices of the greenest/wettest-pixel composite images. The greenest/wettest-pixel composite images selects the pixel with the highest NDVI/LSWI from all the pixels in the growing season, and obtains the corresponding bands and indices (Figs. S2–3). (3) five coefficients of the harmonic regression on the time series of the three indices (NDVI, EVI, and LSWI). We conducted the harmonic regression (discrete Fourier transform) on the original valid observations to extract the temporal characteristics of the time series curves (Eq. 1)^12^.

1 $$V{I}_{t}=c+{a}_{1}{\rm{\cos }}(3\pi t)+{b}_{1}{\rm{\sin }}(3\pi t)+{a}_{2}{\rm{\cos }}(6\pi t)+{b}_{2}{\rm{\sin }}(6\pi t)$$

where *t* means the time of the observation, *VI*_*t*_ refers to the Vegetation Index (VI) at time *t*, *a*_1_, *b*_1,_ *a*_2,_ *b*_2_ and *c* are the five coefficients of the harmonic regression. The *t* is expressed as a fraction between 0 (January 1) and 1 (December 31). In total, 255 feature candidates were prepared for the crop classification (Table 3).

**Table 3:** The summary of the feature candidates for crop type classification.

Group	Periods	Proxies	Metrics	# of features
10-day time series	22 intervals	RE2, SWIR1, SWIR2, NDVI, EVI, LSWI, NDSVI, NDTI, RENDVI, REP	_	220 (10 proxies /interval × 22 intervals)
temporal composite	growing season	RE2, SWIR1, SWIR2, NDVI, EVI, LSWI, NDSVI, NDTI, RENDVI, REP	greenest/wettest-pixel composite*	20 (10 proxies /metric × 2 metrics)
harmonic coefficients	growing season	NDVI, EVI, LSWI	harmonic regression**	15 (3 proxies /metric × 5 metrics)

*greenest/wettest-pixel composite approach selects the pixel with the highest NDVI/LSWI from all the pixels in the growing season, and returns the corresponding proxies (i.e. red2, swir1, swir2, NDVI, EVI, LSWI, NDSVI, NDTI, RENDVI and REP).

**harmonic regression algorithm returns five harmonic coefficients of each of the three proxies (i.e. NDVI, EVI and LSWI).

The classification of the major crops (rice, maize and soybean) in the Northeast China is challenging. The widely used NDVI/EVI time series hardly discriminate the different crop types because these time series overlapped among crops (Fig. S1). Besides the NDVI/EVI time series, we designed a huge size of feature candidates with different spectral domains and temporal windows, which have potential to classify the different crops with a high accuracy (Table 3). The paddy rice could be identified by the 10-day time series of SWIR1, SWIR2, LSWI and NDSVI (Fig. S1). In the flooding/transplanting stage of rice (DOY: 120–150), the reflectance of SWIR1 and SWIR2 of rice was significantly lower than maize and soybean, and the LSWI and NDSVI was correspondingly higher than the other two crops. Maize and soybean could be discriminated by 10-day time series of RENDVI and REP (Fig. S1). In the peak growing stage of these two summer crops (DOY: 200–240), the RENDVI and REP of maize was obviously higher than that of soybean. Additionally, the greenest/wettest-pixel composite images were also useful to discriminate maize and soybean (Figs. S2–3). The value of SWIR1, SWIR2, RENDVI and REP was different among maize and soybean in the greenest/wettest-pixel composite images (Figs. S2–3). Therefore, our hand-crafted feature candidates can identify paddy rice from maize and soybean via the distinct flooding signals in the flooding/transplanting stage of rice. They can also discriminate maize and soybean due to their different reflectance in the shortwave infrared bands and red edge bands in the peak growing stage of these two crops.

Feature selection greatly determine the efficiency of the machine learning algorithms. The optimal subset of hand-crafted features could reduce computational time, especially when dealing with a large volume of images (72,173 images were used in our study). However, it is still unclear which bands or spectral indices would better discriminate crop types. Therefore, we designed a sophisticated feature selection procedure to obtain the optimal cropland/crop features from the large size of feature candidates, based on the two criteria: (1) the important features with high separability among different classes should be retained; (2) the collinearity of each pair of selected features should be relatively low to avoid redundancy^39^. The feature selection procedures were conducted through two steps: First, the feature importance of all the features was assessed by the Mean Decrease Impurity index (MDI), which was calculated by the RF classifier in the scikit-learn python package^40^. The MDI (Gini importance) measures the decrease in the Gini impurity criterion of each feature over all trees in the forest^41^. Considering that accuracies of all the six AGZs reached saturation when the 50 most important features were used, the top 50 features were obtained based on the MDI sorting; Second, the hierarchical clustering of the top 50 features on the Spearman rank-order correlations was performed. The top 50 features were grouped into several clusters via a threshold of the maximum depth, which was set as 1 in this study. One feature with the highest MDI in each cluster was finally kept. In this way, the collinearity of the selected features was significantly decreased. Based on this feature selection procedure, we selected 7–13 optimal features from 184 cropland feature candidates for cropland mapping in the six ACZs (Table S1), and selected 14–25 optimal features from 255 crop feature candidates for crop mapping (Table S2).

### Random Forest algorithm

Random Forests (RF) is an ensemble of decision trees, which were trained based on boot-strap aggregating (bagging) technique. The RF averages the prediction of each individual decision tree to obtain the final prediction. Previous study demonstrated that RF is more robust and accurate than many conventional classifiers, such as maximum likelihood, single decision trees and single-layer neural networks^42^. The RF algorithms in the GEE platform has been successfully used to detect land cover changes^43,44^, to monitor the agricultural land^45^, and to classify the crop types^12^. We adjusted two parameters of RF in the GEE when training the cropland and crop classifiers: (1) numberOfTrees: number of trees determines the number of binary CART trees used to build an RF model. It can be observed that accuracy rises slightly and computational cost increases linearly when the number of trees increases. The numberOfTrees in our study was set to 100 following previous work^13^. (2) minLeafPopulation: The minimum number of samples required to be at a leaf node. We set minLeafPopulation to 10 to limit the depth of each tree to avoid overfitting^13^. The other four parameters, including variablesPerSplit (the number of variables per split, the square root of the number of features by default), bagFraction (the fraction of input to bag per tree, 0.5 by default), outOfBagMode (whether the classifier should run in out-of-bag mode) and seed (random seed), were set by default in the GEE.

## Data Records

Three crop maps with the nominal 10-m resolution are provided for entire Northeast China during 2017–2019. The datasets are available at the figshare repository in a Geotiff format^46^. The dataset is provided in ESPG: 4326 (WGS_1984) spatial reference system. The values of the three crop type maps contains 0,1,2 and 3, representing rice, maize, soybean, and other land (including other crops and non-cropland). The dataset extents from 38.7° N to 53.8° N latitude and 115.5° E to 135.0° E longitude. The maps can be visualized and analyzed in ArcGIS, QGIS, or in similar software.

## Technical Validation

The evaluation of our method and resultant maps includes three aspects: (1) the performance of the RF classifiers in this study was compared with Spectral Angle Mapper (SAM) and Spectral Correlation Mapper (SCM) in Sanjiang plain (SJ) and Songliao plain (SL), the core zones of food production in the Northeast China. The SAM and SCM are two important classification algorithms because they can repress the effects of atmosphere and shading on target reflectance characteristics^47^. Meanwhile, the performance of all feature set and the optimal feature subset was compared to assess the efficacy of feature selection procedure. A total of six scenarios were designed, including: RF with all feature set (RF-All), RF with optimal feature subset (RF-Opt), SAM with all feature set (SAM-Opt), SAM with optimal feature subset (SAM-Opt), SCM with all feature set (SCM-Opt), and SCM with optimal feature subset (SCM-Opt). In each scenario, the half of the training samples in 2018 were used to train the classifiers and to build the reference spectrum, while the rest were used to calculate the overall accuracy (OA). (2) the overall accuracy (OA), user accuracies (UA), producer accuracies (PA), and F1-score (F1) was calculated for the three annual crop maps based on the ground validation samples. There were 8085, 10,658, and 11,035 independent validation samples in 2017, 2018, and 2019. (3) the crop area estimates derived from the annual crop maps were compared with the statistic yearbook at the prefectural level in 2017 and 2018 (absence of 2019 due to the unavailability of the statistical data).

**Fig. 4:**
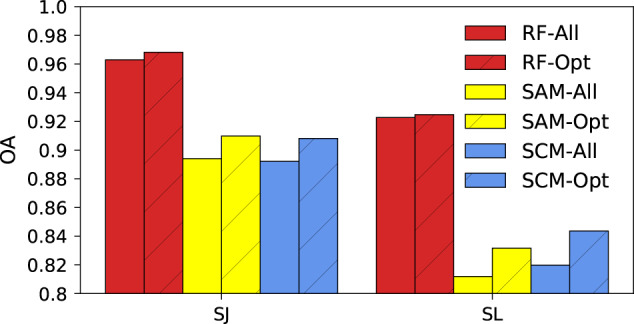
The overall accuracy (OA) of crop classification in Sanjiang plain (SJ) and Songliao plain (SL) in 2018. Six scenarios were included: Random Forest with all feature set (RF-All), Random Forest with optimal feature subset (RF-Opt), Spectral Angle Mapper with all feature set (SAM-Opt), Spectral Angle Mapper with optimal feature subset (SAM-Opt), Spectral Correlation Mapper with all feature set (SCM-Opt), and Spectral Correlation Mapper with optimal feature subset (SCM-Opt).

We found that RF outperformed SAM and SCM in both ACZs (Fig. 4). In average, the OA of RF were 7% and 12% higher than the other two algorithms in SJ and SL, respectively. Meanwhile, the optimal feature subset could generate high accuracy compared with all feature set, with an increasing rate ranging from 0.2% to 3% for all three algorithms and two ACZs. The slight increase of OA might result from the mitigation of the Hughes effect. In summary, the RF algorithm used in this study was superior to SAM and SCM. The feature selection process could not only improve the computing speed, but slightly increase the accuracy.

The OAs of the three crop maps varied from 0.81 to 0.87 (Table 4). Rice was accurately identified with a three-year averaged F1 of 0.93. Soybean and maize had relatively lower accuracies than rice, both with a three-year averaged F1 of 0.83. All user’s accuracy (UA) and producer’s accuracies (PA) of rice in the three years were higher than 0.9 except for the UA in 2017 (0.87). Maize and soybean had higher PA than UA, indicated that the commission errors of maize and soybean were higher than the omission errors. The commission errors of maize and soybean mainly resulted from the incorrect identification of other crops as maize and soybean, which might lead to the overestimation of the planting areas of maize and soybean. In addition, irrigation would change the spectral characteristics of the crop fields, including, but not limited to, greenness, wetness, and thermal properties^19^. Considering the key role of peak growing stage for maize and soybean classification, the different irrigation status in this period might lead to the misclassification among maize and soybean.

**Table 4:** The confusion matrix of the crop type maps based on sufficient ground truth data from 2017 to 2019. Map categories are rows while reference categories are columns.

Year	Crop	rice	maize	soy	others	Users accu.	F1	Overall accu.
2017	rice	868	101	9	20	0.87	0.89	0.81
	maize	15	1783	65	441	0.77	0.81	
	soy	17	126	2210	469	0.78	0.84	
	others	44	81	180	1660	0.84	0.73	
	Producers accu.	0.92	0.85	0.90	0.64			
2018	rice	1206	11	1	39	0.96	0.96	0.81
	maize	9	2241	67	667	0.75	0.82	
	soy	3	121	2197	864	0.69	0.79	
	others	46	86	131	2974	0.92	0.76	
	Producers accu.	0.96	0.91	0.92	0.65			
2019	rice	1092	57	3	16	0.94	0.94	0.87
	maize	5	2380	39	419	0.84	0.85	
	soy	6	111	2282	324	0.84	0.87	
	others	53	229	203	3821	0.89	0.86	
	Producers accu.	0.94	0.86	0.90	0.83			

**Fig. 5:**
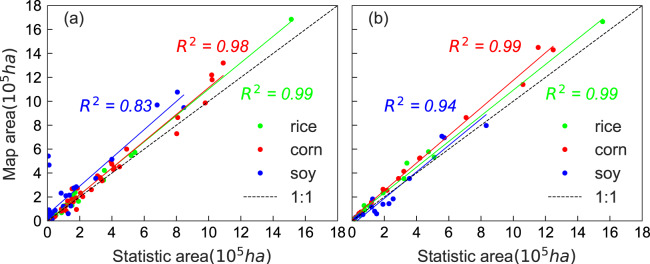
The comparison of the estimated planting area of rice, maize, and soybean from our annual crop maps with the statistical data at the municipal level in 2017 (**a**) and 2018 (**b**).

The area estimates derived from our crop maps were compared with the statistical data in the yearbook at the prefectural level in 2017 and 2018 (Fig. 5). The area of rice from the crop maps was highly related to the statistical data in both years, with R^2^ of 0.99. The area of maize was also very consistent with the statistical data, with R^2^ of 0.98 and 0.99 in 2017 and 2018, respectively. The area of soybean was less correlated with the statistical data than rice and maize, with R^2^ of 0.83 and 0.94 in 2017 and 2018, respectively. The estimated area of soybean in the Baicheng municipality and the Songyuan municipality had relatively higher bias compared with the statistical data. According to the statistical data, the area of soybean occupied less than 1% of the total crop planting area in these two municipalities. The planting areas of maize, rice, oil plants, sunflower, and vegetables were higher than that of soybean. Some peanuts, sunflowers, and vegetables were falsely mapped as soybean, causing the potential overestimation of the minority soybean planting area.

## Usage Notes

**Fig. 6:**
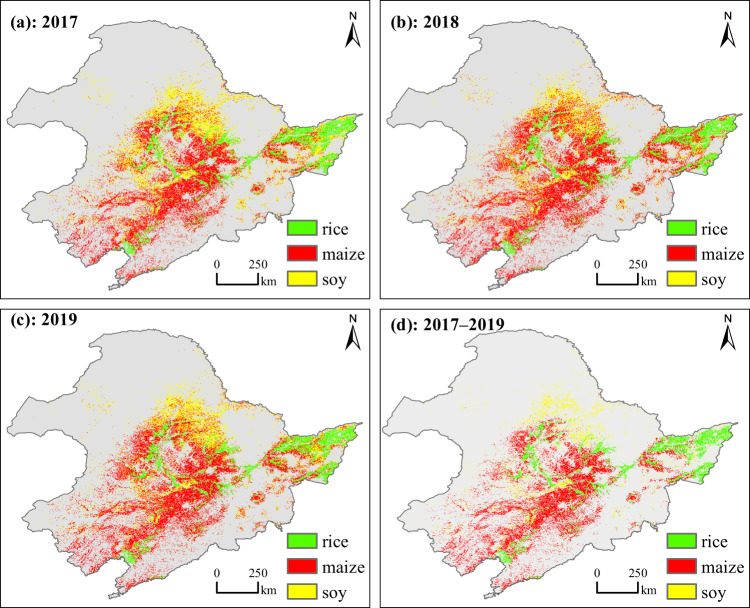
The crop maps in Northeast China in 2017 (**a**), 2018 (**b**), and 2019 (**c**), and the unchanged rice, maize and soybean during 2017–2019 (**d**).

**Fig. 7:**
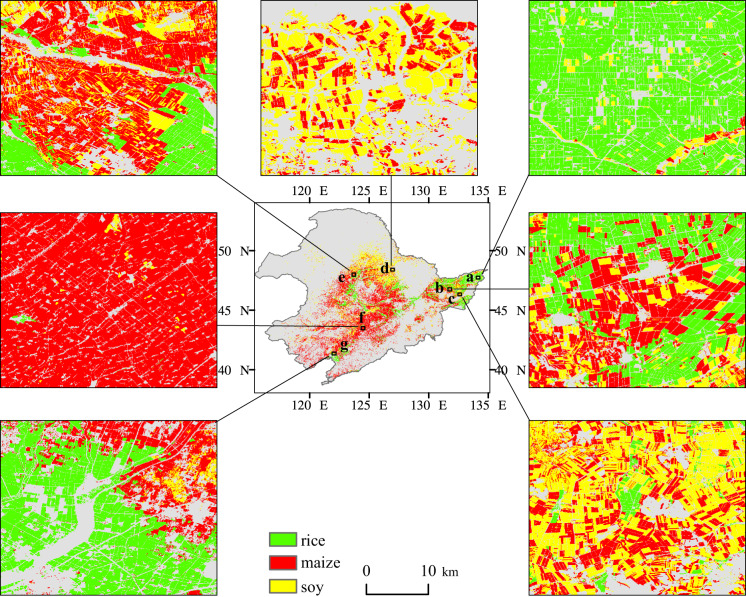
The spatial details of the crop map in 2019 in Northeast China. Site a (134.2° E, 47.7° N), b (131.8° E, 46.7° N) and c (132.6° E, 46.3° N) were located in the Sanjiang Plain (SP); Site d (127.0° E, 48.4° N), e (123.7° E, 48.0° N) and f (124.5° E, 43.5° N) were located in the Songliao Plain (SL); Site g (122.1° E, 41.3° N) was located in the Liaodong Peninsula (LD).

The information on the crop planting areas in Northeast China, one of the most important food bowl in China, is vital for understanding the regional and national food security, in the context of continuously growing population and consumption^48^. In this study, we provided major crop type maps with a 10-m resolution during 2017–2019 (Fig. 6). This spatially explicit crop maps can be used to support crop yield and production forecasting at the parcel level when combining with crop models (Fig. 7)^49^. This dataset can also be used to support related studies on regional water use, soil fertility, and land degradation in the Mollisol region of Northeast China^50,51^. The annual crop maps can also provide quantitative information about the changes in the farming system, which is vital to assess the performance of the soybean rejuvenation plan and crop rotation incentive policy^2^. To track the long-term changes in the crop planting area, a valuable extension to the present dataset would be the inclusion of the historical crop type maps before 2017, which might be achieve by retrospectively map crop cover history using the Landsat and MODIS archive.

## Supplementary information

Supplementary Material

## Data Availability

JavaScript code used to generate the cropland layer and crop type maps are available from the figshare repository^46^.
