# Supplementary material for: The 10-m crop type maps in Northeast China during 2017–2019
Source: Sci Data. 2021 Feb 2;8:41. doi: 10.1038/s41597-021-00827-9 (PMC7854749; doi:10.1038/s41597-021-00827-9)
Supplement: Supplementary file 1 — Supplementary Material [file 41597_2021_827_MOESM1_ESM.docx]

Supplementary Material for

**The 10-m crop type maps in Northeast China during 2017-2019**

Nanshan You^1,2^, Jinwei Dong^1^*, Jianxi Huang^3^, Guoming Du^4^, Geli Zhang^3^, Yingli He^1^, Tong Yang^3^, Yuanyuan Di^3^, and Xiangming Xiao^5^*

^1^Key Laboratory of Land Surface Pattern and Simulation, Institute of Geographic Sciences and Natural Resources Research, Chinese Academy of Sciences, Beijing 100101, China;

^2^University of Chinese Academy of Sciences, Beijing 100049, China;

^3^College of Land Science and Technology, China Agricultural University, Beijing 100083, China;

^4^School of Public Administration and Law, Northeast Agricultural University, Harbin 150030, China;

^5^Department of Microbiology and Plant Biology, University of Oklahoma, Norman, OK 73019, USA.

Corresponding author(s): Jinwei Dong ([dongjw@igsnrr.ac.cn](mailto:dongjw@igsnrr.ac.cn)); Xiangming Xiao ([xiangming.xiao@ou.edu](mailto:xiangming.xiao@ou.edu))

**Table of contents**

[Figure S1. 2](#_Toc58356906)

[Figure S2. 3](#_Toc58356907)

[Figure S3. 3](#_Toc58356908)

[Table S1 3](#_Toc58356909)

[Table S2 4](#_Toc58356910)


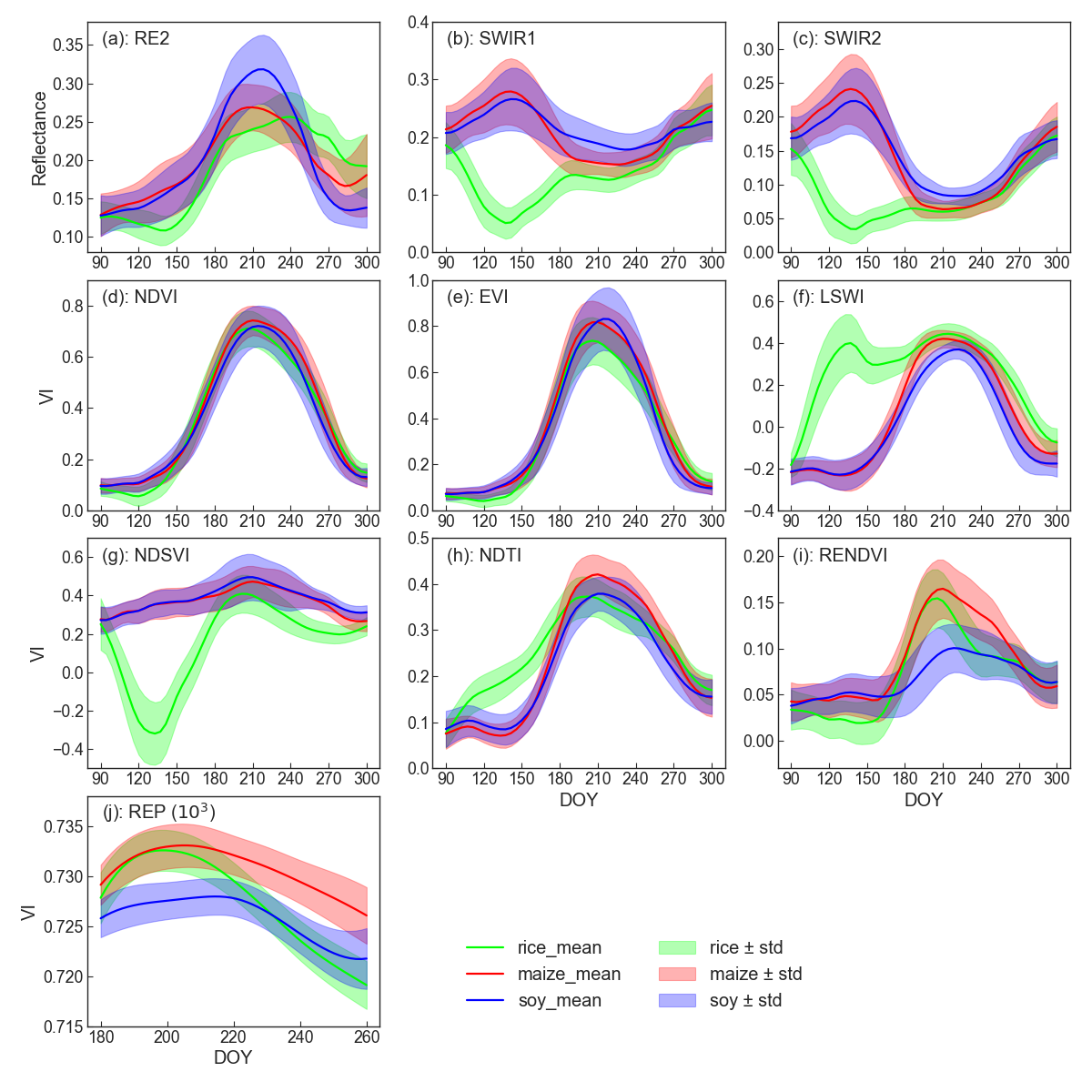


Figure S1. The 10-day time series of the reflectance of the three reflectance bands (RE2, SWIR1, and SWIR2) and seven spectral indices (NDVI, EVI, LSWI, NDSVI, NDTI, RENDVI, and REP) during DOYs 90-300. The mean values and standard deviation (std) of each value in the each 10-day interval were shown based on the training samples in the Sanjiang Plain (SJ) in2018. REP values before the DOY of 180 and after the DOY of 260 were not showed due to the large variabilities in these periods.


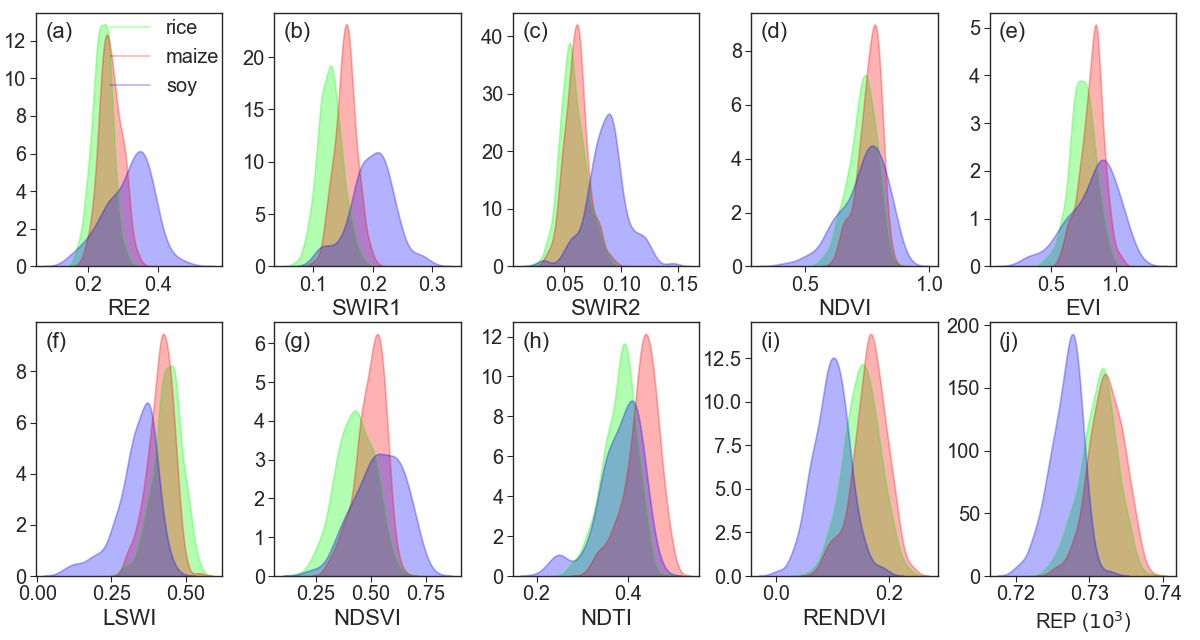


Figure S2. The probability density curve of the greenest-pixel composited pixels of rice, maize and soybean in the Sanjiang (SJ) plain in 2018.


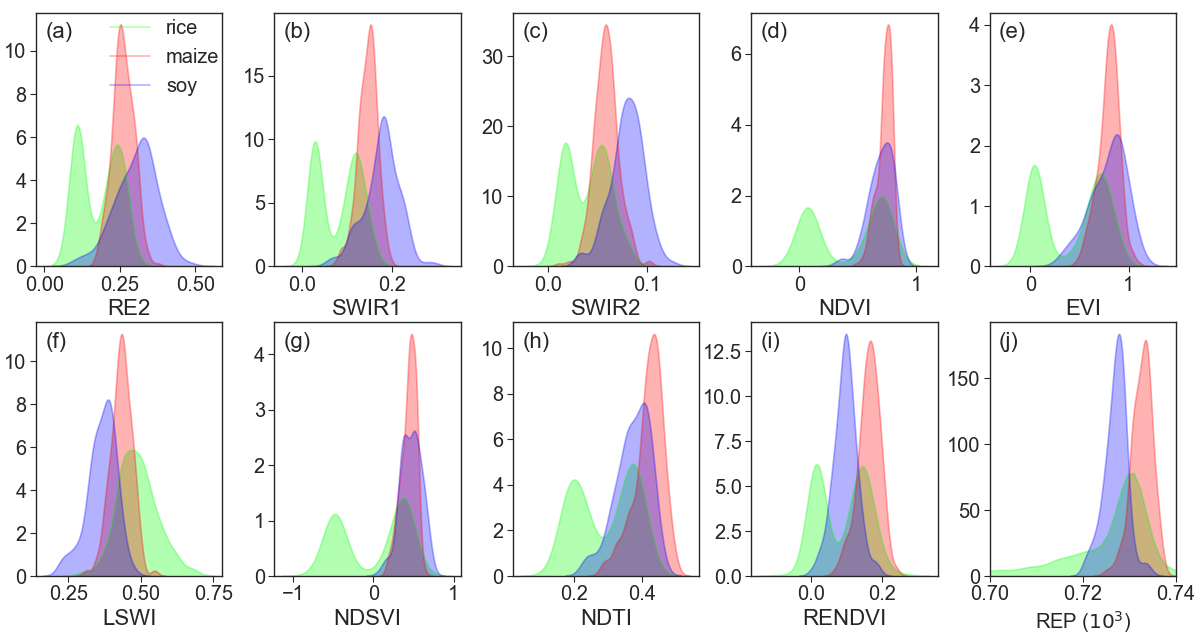


Figure S3. The probability density curve of the wettest-pixel composited pixels of rice, maize and soybean in the Sanjiang (SJ) plain in 2018.

Table S1 The optimal cropland features in each agro-climate zone (ACZ) based on the feature selection procedure. The median composites in the crop seeding, growth, and harvest stage were labelled as _1, _2, and _3; the minimum, maximum, mean, standard deviation and amplitude of the observations in the growing season were labelled as _min, _max, _mean, _std and _amp; the 5th, 25th, 50th, 75th, and 95th percentiles of the observations were labelled as _p5, _p25, _p50, p75, and p95.

| ACZ | Optimal cropland features | # of features |
| --- | --- | --- |
| GK | SWIR2_p95, NDTI_p25, SWIR2_p75, NDTI_mean, NDTI_p5, NDTI_std, NDSVI_max | 7 |
| SJ | NDVI_1, NDVI_std, NDVI_mean, EVI_std, NDVI_p95, REP_p95, NDVI_p5, REP_p75, EVI_p50, NDTI_mean, RENDVI_3, NDTI_1, SWIR1_std | 13 |
| LK | SWIR2_std, NDVI_1, NDTI_amp, NDTI_p5, NDVI_std, LSWI_1, REP_2, EVI_std, RENDVI_p75, NDVI_p95 | 10 |
| SL | NDTI_amp, NDVI_std, NDVI_1, NDTI_p5, REP_p75, LSWI_1, NDVI_p95, SWIR2_std, NDSVI_max, NDVI_mean | 10 |
| LD | SWIR2_std, NDTI_std, NDVI_std, SWIR2_p95, NDTI_p5, LSWI_1, NDVI_p25, SWIR2_1, REP_2, slope, EVI_max | 11 |
| IM | NDTI_amp, SWIR2_std, REP_p75, RENDVI_amp, NDVI_std, NDTI_1, LSWI_p95 | 7 |

Table S2 The optimal crop features in each agro-climate zone. The number followed the bands or indices referred to the Day of Year (DOY); the greenest/wettest-pixel composite values were labelled as _NDVI and _LSWI; the five coefficients of the harmonic regression were labelled as _constant, _cos3, _sin3, _cos6, and _sin6.

| ACZ | Optimal crop features | # of features |
| --- | --- | --- |
| GK | NDVI_230, RE2_220, NDVI_240, RE2_210, NDVI_cos6, EVI_sin6, LSWI_230, SWIR1_190, LSWI_180, SWIR2_190, REP_190, RENDVI_190, REP_NDVI, NDVI_160, NDVI_170, NDVI_150, LSWI_NDVI, LSWI_220, SWIR1_260, NDVI_NDVI, RENDVI_200, LSWI_170, RE2_190, LSWI_210, EVI_sin3 | 25 |
| SJ | SWIR1_150, LSWI_130, SWIR2_140, LSWI_150, REP_200, REP_210, REP_LSWI, REP_220, REP_230, REP_250, RENDVI_NDVI, SWIR2_NDVI, LSWI_190, NDTI_130, RENDVI_LSWI, REP_180, LSWI_210, SWIR1_NDVI, LSWI_180 | 19 |
| LK | REP_200, LSWI_160, NDSVI_150, REP_220, SWIR2_150, LSWI_150, SWIR1_170, REP_250, NDSVI_140, RENDVI_NDVI, SWIR1_140, SWIR1_NDVI, LSWI_180, REP_180, RENDVI_240, SWIR1_180, SWIR2_210, SWIR2_200, SWIR1_240 | 19 |
| SL | REP_240, REP_210, REP_250, SWIR2_150, NDSVI_150, NDSVI_140, RENDVI_220, LSWI_140, RENDVI_230, LSWI_160, SWIR2_170, SWIR1_210, RE2_220, NDSVI_260 | 14 |
| LD | LSWI_140, SWIR1_150, LSWI_sin6, NDSVI_160, RE2_150, EVI_140, LSWI_280, EVI_sin6, NDVI_cos3, LSWI_cos3, SWIR1_140, REP_LSWI, LSWI_210, RE2_NDVI, REP_NDVI, LSWI_constant, RE2_250, REP_210, EVI_290, RE2_200, LSWI_240, SWIR1_220 | 22 |
| IM | REP_210, REP_240, RENDVI_220, RE2_NDVI_max, REP_200, RENDVI_210, RE2_230, NDSVI_300, RE2_210, EVI_sin6, NDSVI_150, EVI_cos3, NDVI_150, LSWI_260, NDVI_140, NDVI_160, LSWI_280, NDTI_140, NDVI_220, REP_180, EVI_230 | 21 |
